# Exploring GP and patient attitudes towards the use and deprescribing of dietary supplements: a survey study in Switzerland

**DOI:** 10.1186/s12875-024-02605-z

**Published:** 2024-10-03

**Authors:** Renata Vidonscky Lüthold, Zsofia Rozsnyai, Kristie Rebecca Weir, Sven Streit, Katharina Tabea Jungo

**Affiliations:** 1 Institute of Primary Health Care (BIHAM), University of Bern, Bern, 3012 Switzerland; 2 Graduate School for Health Sciences, University of Bern, Bern, 3012 Switzerland; 3 Sydney School of Public Health, Faculty of Medicine and Health, University of Sydney, Sydney, 2050 Australia; 4 Center for Healthcare Delivery Sciences, Brigham and Women’s Hospital, Boston, MA 02115 United States of America; 5 Division of Pharmacoepidemiology and Pharmacoeconomics, Department of Medicine, Brigham and Women’s Hospital, Harvard Medical School, Boston, MA 02115 United States of America

**Keywords:** Primary care, Dietary supplements, Deprescribing, Polypharmacy, Older adults, Patient preferences

## Abstract

**Background:**

Dietary supplements are commonly used by older adults, but their inappropriate use may lead to adverse events. To optimise medication use, general practitioners (GPs) ideally are aware of all substances that patients use, including supplements. This cross-sectional study explored the use of dietary supplements by older patients with polypharmacy, the rate at which they disclosed this use to their GPs, and compared patients’ and GPs’ attitudes towards discontinuing dietary supplements.

**Methods:**

Ten GPs in Swiss primary care recruited five to ten of their older patients taking ≥ 5 regular medications. Both GPs and their patients completed a survey on patients’ use of dietary supplements and attitudes towards deprescribing those. We described and compared their responses. We assessed the association of supplement disclosure with patient characteristics using multilevel logistic regression analysis.

**Results:**

Three out of ten GPs (30%) were female, and GPs’ average age was 52 years (SD = 8). 45% of patients were female (29/65). Most patients (*n* = 45, 70%) were taking ≥ 1 supplement. On average, patients reported to be using three supplements (SD = 2). In 60% (*n* = 39) of patients, GPs were unaware of ≥ 1 supplement used. We did not find evidence for an association between supplement disclosure to GPs and patient characteristics. Only 8% (*n* = 5) of patients and 60% (*n* = 6) of GPs reported ≥ 1 supplement they would be willing to deprescribe and none of the supplements reported by GPs and patients to deprescribe matched.

**Conclusion:**

Swiss GPs were unaware of many dietary supplements used by their older patients, which may affect medication optimisation efforts.

**Supplementary Information:**

The online version contains supplementary material available at 10.1186/s12875-024-02605-z.

## Background

Dietary supplements are defined as products containing vitamins, minerals, herbs or other botanicals, amino acids and/or other dietary substances used to supplement a person’s diet [1, 2]. The use of supplements is common and has been increasing in older adults, despite the underreporting of use in health surveys [3–8]. In Switzerland, the prevalence of dietary supplement use among the adult population was between 26% and 53% from 2009 to 2023 [6, 9, 10]. According to the annual report of a Swiss health insurance, vitamin D was one of the most commonly purchased substances in 2021 and the use of vitamin B12 increased by 36% from 2021 to 2022 [11, 12]. In Switzerland, select supplements prescribed by physicians (such as vitamin D and vitamin B12) are covered by the mandatory health insurance. Despite the widespread use of supplements, there are still gaps regarding the use and attitudes towards dietary supplements of older adults with polypharmacy.

Dietary supplements are often associated with a healthier life and are perceived as being risk-free [13–16]. Indeed, dietary supplements are important for vulnerable groups at risk for nutritional deficiency, but the benefits of taking many supplements without a clear indication are inconclusive for older adults [17–19]. Given the low-risk perception towards dietary supplements and their easy accessibility, many patients do not disclose their use of supplements to their general practitioners (GPs) and other healthcare providers [5, 14, 16, 17, 20]. However, dietary supplements may also lead to adverse events, drug interactions, and hospital admissions, especially in older adults in which drug metabolism is compromised [19, 21, 22]. Furthermore, dietary supplements are often used when there is a lack of indication [3–5, 23]. When an indication for the use of the dietary supplement is not present, these are only contributing to patients’ pill burden and present an unnecessary financial burden [14, 24]. Dietary supplements contribute to polypharmacy (use of ≥ 5 medications) and might be easy targets for deprescribing (stopping or reducing substances that are non-longer needed [25–27]) [14, 28]. However, there is a lack of studies focusing on provider and patient attitudes towards deprescribing potentially inappropriate supplements [14, 29].

When GPs are unaware of patients’ use of dietary supplements, they may not associate patients’ symptoms with adverse effects caused by supplements. Instead, they may approach these symptoms as a new condition, start a new drug to treat it, and thereby initiate a prescribing cascade and contributing to polypharmacy [30, 31]. Therefore, GPs should not only consider prescription medications, but also supplements when carrying out medication reviews and deprescribing efforts. To be able to identify and manage patients at risk of potentially inappropriate use of supplements, GPs and other healthcare providers must be informed about which supplements patients are using [5, 32]. However, there is limited information on how often older adults disclose their supplement use to their healthcare providers [5, 14, 17].

In addition, although most of the research on deprescribing focuses on prescription medications only, when healthcare professionals are asked which medications they would be more willing to stop or reduce in their patients, dietary supplements are often mentioned and seem to have promising deprescribing outcomes [28, 33]. However, these studies are focused on overall willingness to deprescribe, considering prescription and non-prescription medications, with little information focusing on patients’ and GPs’ willingness to stop or reduce dietary supplements. Studies focusing on overall willingness to deprescribe are limited by the lack of information on whether all prescribers accounted for all supplements when making deprescribing decisions. For instance, in different settings (e.g., different countries), dietary supplements might be part of patients’ medication list, but in others, they are not. Supplements that are not prescribed are not always included in medication lists. With this, little is known about how patient and GP attitudes towards deprescribing supplements compare.

In this context, we aimed to investigate (a) the use, beliefs, and motivations of older patients with polypharmacy for using dietary supplements, (b) the rate at which the use of supplements is disclosed to GPs by patients, and (c) to explore and compare older patients’ and their GPs’ attitudes towards deprescribing dietary supplements.

## Materials and methods

### Settings and study design

This cross-sectional study is part of the “Understanding older patients’ willingness to have medications deprescribed in primary care” study [34]. GPs practising in the German-speaking part of Switzerland were invited to participate. The inclusion criteria for GPs were to be actively working in primary care in the German-speaking part of Switzerland. Each GP was asked to consecutively recruit a sample of five to ten of their primary care patients aged ≥ 65 years old with polypharmacy (≥ 5 long-term medications). The recruitment took place from May 2022 to November 2023. Written informed consent was documented for each patient. More details on the study design have been described in the study protocol [34]. The competent ethics committee in Switzerland (*Kantonale Ethikkommission Bern*) approved the present study in January 2022 (Project-ID 2022–00035).

### Data source and data collection

GPs and their recruited patients were invited to complete a survey, which could be completed either online or on paper. For paper surveys, patients had four weeks to return the completed questionnaire in a sealed envelope to their GP practice. GPs then returned the sealed envelopes from patients and their own (which they had completed independently for each of the recruited patients) to the research team at the University of Bern. A study team member then entered the data into the REDCap study database [35, 36].

### Questionnaire

The content of the survey was based on the literature and clinical rationale [7, 13, 37–39]. Each GP received one survey for each one of their recruited patients. The GP survey contained questions on background information (e.g., age, gender, years of work experience), dietary supplements disclosed to be taken by or prescribed to each of the recruited patients, and attitudes towards deprescribing those. The survey for patients contained questions on sociodemographic characteristics, use of dietary supplements, attitudes and beliefs towards dietary supplements, attitudes towards having those deprescribed and trust in their physician [40]. Trust scores were calculated using the five 5-point Likert scale questions from Dugan et al., 2005, in which total scores range from 5 to 25, with higher scores representing higher trust in the physician [40]. The questionnaire was piloted with five older adults before the start of the data collection, which helped shorten the questionnaire and enhanced its clarity. The English version of the questionnaires can be found in the supporting material (Additional File 1 and Additional File 2).

### Variables and data management

To be able to identify patient-GP dyads, GPs reported their names and the patients’ names in each questionnaire. Patients on the other hand provided their names in the informed consent form attached to the survey and reported their GP’s name in the survey. Identifiable variables (GPs’ and patients’ names) were only used to certify the merging of the questionnaires and deleted afterwards.

### Outcomes

#### Patient-reported supplement use

We assessed the use of supplements reported by the patient using the question ‘*Do you regularly take vitamins*, *mineral or herbal supplements?’.* To assess which dietary supplements patients were using, each patient received a list of 24 commonly used dietary supplements, from which they could choose which one(s) they were currently using (see Additional File 1). In addition, patients were able to report additional supplements as free text by choosing the option ‘*other*’. Dietary supplements reported in the free text were categorised according to the supplement substance (e.g., vitamin C, Iron, Calcium, Valerian, etc.) and defined as vitamins, minerals, amino acids, essential fatty acids, plants and/or herbal extracts [1, 2]. For feasibility reasons, patients could choose a maximum of three dietary supplements for which they could give more information on the reasons why they are using these supplements and who recommended these supplements to them.

#### GP reported supplement use

GPs received the same list of 24 dietary supplements. Based on their knowledge, GPs selected the supplements they believed their patients were using. GPs were able to report additional supplements as free text by choosing the option ‘*other*’ (see Additional File 2).

#### Disclosure of supplement use

To explore whether patients disclosed their dietary supplement use to their GPs, we compared the responses of GPs and patients. Disclosure was defined as follows: (i) when both the GP and the patient reported the same supplements, we assumed that the supplement use was disclosed (i.e., the GP knew about the supplement taken); (ii) when the supplement was reported by either the patient or the GP, we assumed that the supplement use had not been disclosed.

#### Willingness to stop/reduce supplement use

For each recruited patient, GPs reported the supplements they would be willing to deprescribe as free text. Patients reported the supplements they would be willing to deprescribe by responding to the 5-point Likert scale question *‘I would be willing to stop or reduce the dosage of this supplement’* for each supplement they were currently taking. These responses were dichotomised considering the options ‘*strongly agree’* and *‘agree*’ as willingness to deprescribe each specific supplement. Willingness to deprescribe was considered for individual supplements.

#### Agreement to stop/reduce

When GPs and patients chose the same supplement for deprescribing, we considered this as agreement to deprescribe. When GPs and patients mentioned different supplements, we considered it as disagreement.

### Statistical analysis

We used descriptive statistics to report patient and GP characteristics, the frequency and reasons for using dietary supplements, and the beliefs of older adults regarding dietary supplements. Continuous variables were presented as means and standard deviations (SD) and categorical variables as frequencies and percentages. We compared the opinions on dietary supplements between users and non-users using the chi-square test and Fischer’s exact test. To analyse the association between the disclosure of the use of dietary supplements with patient characteristics, we performed a multilevel logistic regression at the supplement level, accounting for clusters at the GP and patient levels. We first based the selection of covariates in the regression model on clinical rationale and literature [14, 41, 42]. We then assessed multicollinearity between variables, which left us with the following covariates: patient gender, education, trust in the physician, and supplement type. To investigate and compare the willingness of patients and their GPs to stop or reduce the use of dietary supplements, we used descriptive statistics (numbers and percentages). To assess the deprescribing agreement, we described the number and percentage of situations in which patients and GPs agreed. We handled missing data by conducting sensitivity analyses, restricting, and comparing responses of those who did and who did not fully complete the questionnaire, and identifying missingness at random for all these variables. We used a complete case analysis to treat missing data. We used Stata 16.1 (StataCorp, College Station, TX, USA [43]) to perform the analysis. A two-sided *p*-value of 0.05 was considered statistically significant.

## Results

We collected data from ten GPs (three were (30%) female, mean age was 52 years (SD = 8)) and 65 of their patients (29 (45%) female, average of seven patients per GP (range: 4 to 16)). Table 1 shows the sociodemographic characteristics of patients and GPs. Most patients had at least secondary school level (*n* = 42, 65%), 48% (*n* = 31) reported making ends financially quite easily, 89% (*n* = 58) were born in Switzerland, 58% (*n* = 38) self-rated their overall health as good or excellent, and 53% (*n* = 35) were quite or extremely confident in filling out medical forms. The score of the abbreviated Wake Forest Trust in Physician Scale [40] was 22 (SD = 4, range: 5–25, with higher values indicating higher trust). 51% (*n* = 33) of the patients had been seeing their current GP for more than 9 years. Most patients (*n* = 45, 70%) reported to be taking at least one dietary supplement, and the average number of supplements taken by patient was three (SD = 2). Of the 45 patients using dietary supplements, 67% (*n* = 30) responded that they talk to the GP or pharmacist before taking any dietary supplement, and 67% (*n* = 43) of the overall sample agreed or strongly agreed that they should speak to their GP, a pharmacist, or another health professional before using any supplement (Table 1).

**Table 1:** Patient characteristics according to patients’ reported use of dietary supplements

Patient characteristics	Total (*n* = 65), *n (%)*	Use of dietary supplements (*n* = 45) ^£^	No use of dietary supplements (*n* = 18) ^£^
Female gender, *n* (%) ^a^	29 (45%)	25 (56%)	3 (17%)
What is your highest completed education?			
None, *n* (%)	2 (3%)	2 (4%)	0
Primary school, *n* (%)	20 (31%)	14 (31%)	4 (22%)
Secondary school, *n* (%)	33 (51%)	23 (51%)	10 (56%)
Third level education, *n* (%)	9 (14%)	5 (11%)	4 (22%)
How do you make ends financially?			
Without any problem, *n* (%)	26 (40%)	20 (44%)	5 (28%)
Quite easily, *n* (%)	31 (48%)	20 (44%)	10 (56%)
With some difficulty, *n* (%)	6 (9%)	3 (7%)	3 (17%)
With great difficulty, *n* (%)	1 (2%)	1 (2%)	0
Born in Switzerland, *n* (%)	58 (89%)	39 (87%)	18 (100%)
In general, how would you describe your health today?			
Excellent, *n* (%)	2 (3%)	1 (2%)	1 (6%)
Good, *n* (%)	36 (55%)	25 (56%)	10 (56%)
Average, *n* (%)	21 (32%)	15 (33%)	6 (33%)
Poor, *n* (%)	4 (6%)	2 (4%)	1 (6%)
How confident are you filling out medical forms by yourself?			
Extremely, *n* (%)	12 (18%)	2 (4)	2 (11%)
Quite a bit, *n* (%)	23 (43%)	4 (9)	11 (61%)
Somewhat, *n* (%)	16 (25%)	13 (29%)	3 (17%)
A little bit, *n* (%)	7 (11%)	4 (9%)	2 (11%)
Not at all, *n* (%)	2 (3%)	2 (4%)	0
How many different medications do you use regularly?			
Mean (SD)	7 (2)	7 (2)	6 (2)
Trust in the physician, mean (SD) ^b^	22 (4)	22 (5)	22 (3)
How long have you been seeing your GP?			
0–9 years, *n* (%)	30 (46%)	21 (47%)	9 (59%)
10–19 years, *n* (%)	18 (28%)	14 (31%)	4 (22%)
20–29 years, *n* (%)	13 (20%)	9 (20%)	4 (22%)
30 + years, *n* (%)	2 (3%)	0	0
Number of dietary supplements used, mean (SD)	-	3 (2)	-
Talk to GP or pharmacist about taking dietary supplements, *n* (%)	-	30 (67%)	-
I should speak to my GP/pharmacist/another health professional before taking any herbal, vitamin, or mineral supplement.			
Strongly disagree, *n* (%)	1 (2%)	1 (2%)	0
Disagree, *n* (%)	5 (8%)	2 (4%)	3 (17%)
I do not know, *n* (%)	8 (12%)	4 (9%)	3 (17%)
Agree, *n* (%)	27 (42%)	18 (40%)	9 (50%)
Strongly Agree, *n* (%)	16 (25%)	14 (31%)	1 (6%)

SD, Standard deviation

^£^ 3 (5%) missing responses on the question *“Do you regularly take vitamins*, *mineral or herbal supplements?”*

For all variables presented the missingness was 0. Exceptions: Gender, higher education, financial status, birth country: *n* = 1 (2%); Patient health status, time seeing the GP: *n* = 2 (3%); Number of medications: *n* = 4 (6%); ‘I should speak to my GP […]’ and Trust: *n* = 8 (12%)

^a^ None of the participants chose the option “other” for gender

^b^ Score of the abbreviated Wake Forest Trust in Physician Scale [40]. Score is within 5 to 25, with higher values indicating higher trust

Patients reported to be taking on average seven (SD = 3) prescription medications, while GPs reported that patients were taking on average nine (SD = 3) prescription medications (Table 1). GPs had on average 15 years of work experience as a GP (SD = 6) (data not shown). GPs reported that they recommended dietary supplements for 52% (*n* = 31) of the patients and responded to be aware that 63% (*n* = 41) of the patients were currently using ≥ 1 dietary supplement (data not shown). Supplements most commonly used were vitamin D, vitamin B12, and magnesium. Of the patients using dietary supplements, 44% (*n* = 20) reported buying their dietary supplements at a pharmacy^2^, 18% (*n* = 8) at the supermarket, 18% (*n* = 8) at the GP practice^1^, 8% (*n* = 4) at the drugstore^2^, 5% (*n* = 2) on the internet, 2% (*n* = 1) at the health food store (data not shown). Of the patients taking supplements, 75% (*n* = 34) said that the supplement was recommended by their GP, 31% (*n* = 14) that they decided to take it themselves, and 20% (*n* = 9) that it was recommended by another physician (data not shown).

**Fig. 1:**
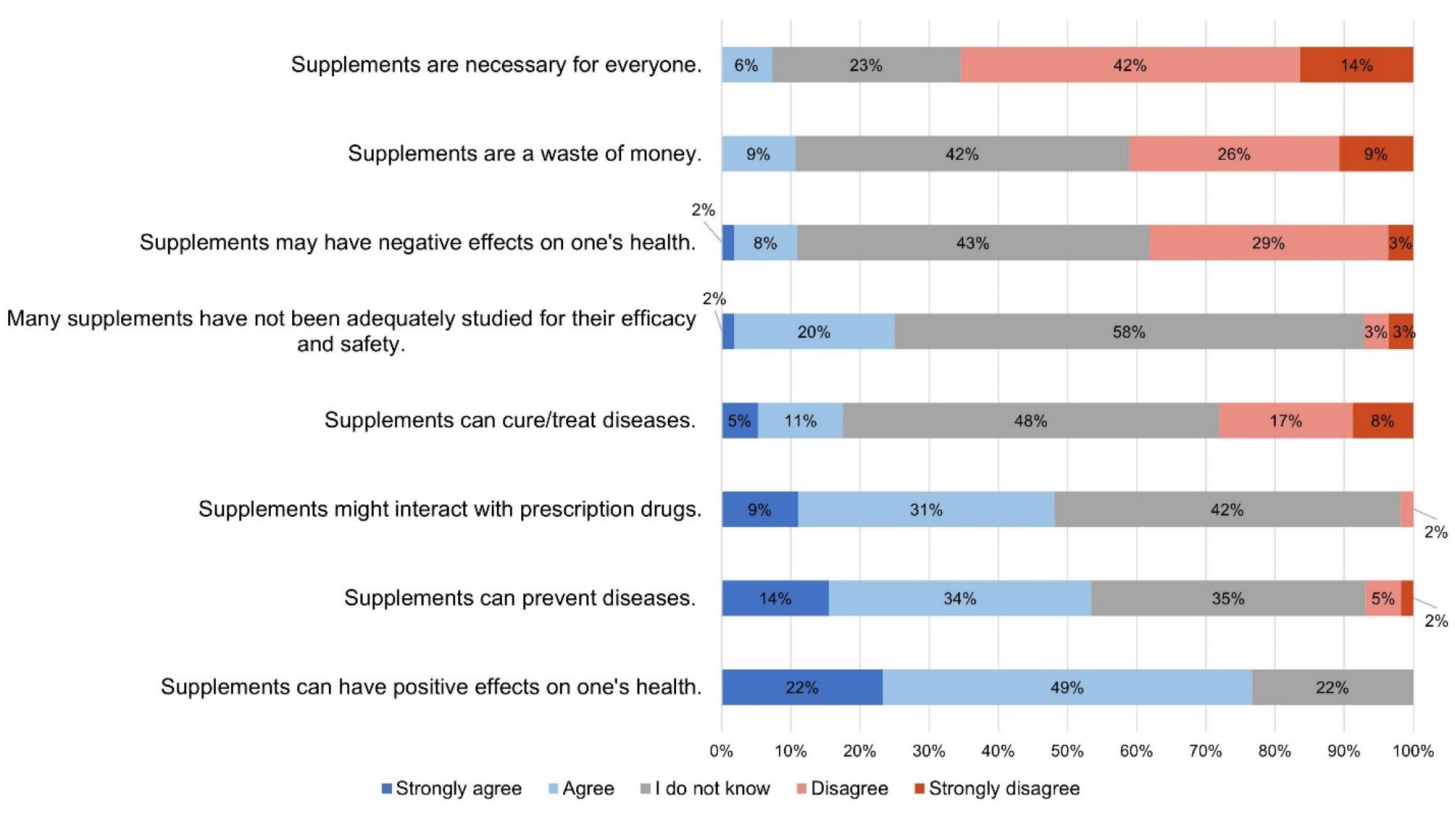
Perceptions towards dietary supplements in older patients with polypharmacy (*n* = 65)

Patient attitudes towards dietary supplements are shown in Fig. 1. For most of the statements, most patients were unsure about their beliefs. Regarding benefits, 71% (*n* = 46) of patients agreed or strongly agreed that supplements are beneficial and 48% (*n* = 31) that they may prevent diseases. Despite these positive beliefs, only 6% (*n* = 4) of the patients believed that everyone needs dietary supplements. Regarding risk perception towards dietary supplements, 40% (*n* = 26) believe that supplements can interact with other drugs. Additional File 3 - Figure S1 shows the agreement with each statement among users and non-users of dietary supplements. 75% (*n* = 34) of users versus 61% (*n* = 11) of non-users agreed or strongly agreed that supplements can have positive effects on one’s health, 49% (*n* = 22) of users and 39% (*n* = 7) of non-users believed that supplements may prevent diseases, and 17% (*n* = 8) of users versus 6% (*n* = 1) of non-users believed that supplements treat diseases. Comparing the agreement with each statement between users and non-users, we did not find any significant difference (Additional File 3 – Table S1).

**Fig. 2:**
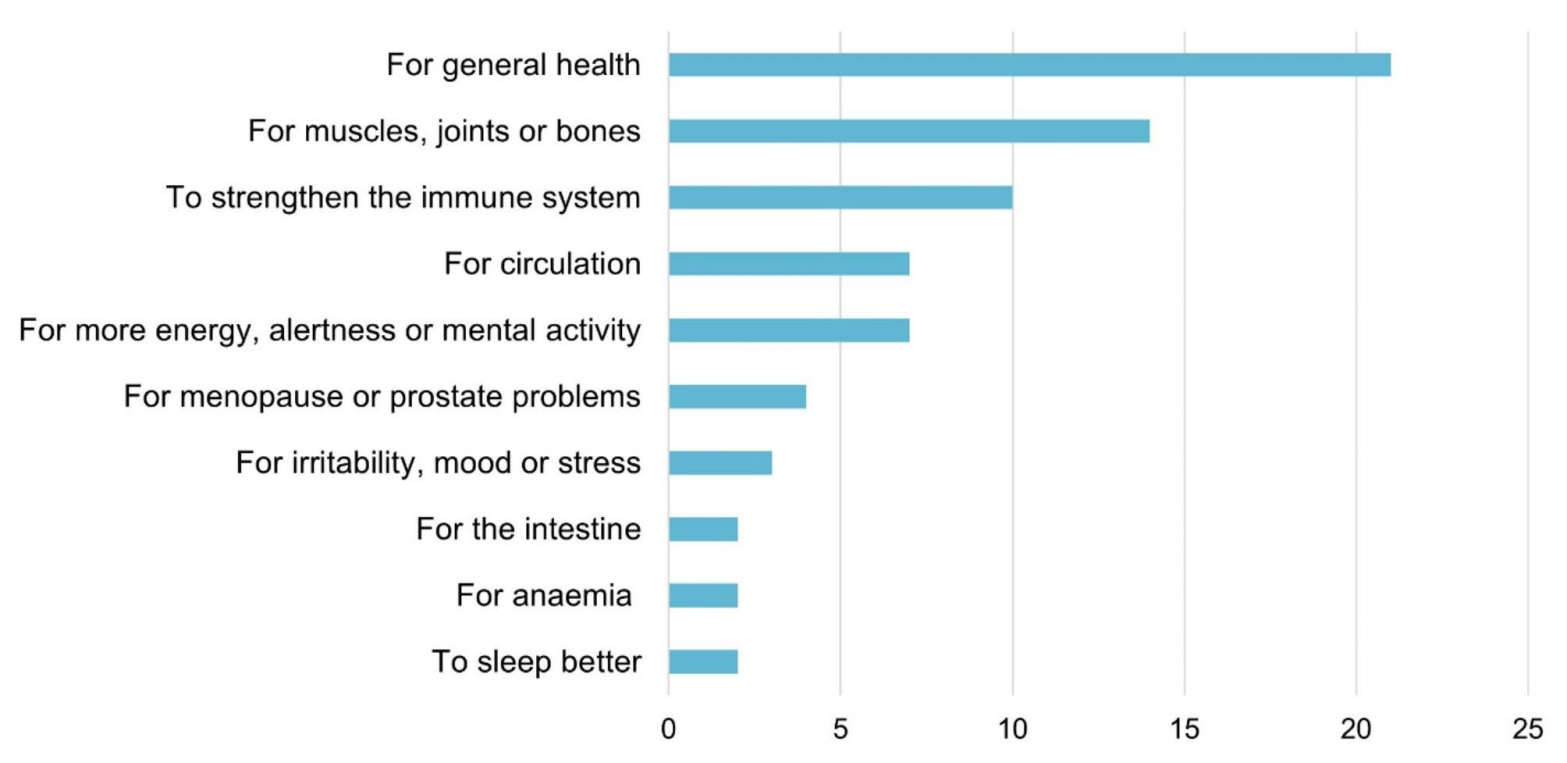
Reasons why older adults with polypharmacy living in the German part of Switzerland use dietary supplements (*n* = 45) Multiple answers were possible

The three most common supplement categories reported by patients were vitamins minerals, and herbs. The dietary supplements most frequently mentioned by GPs were vitamin D (*n* = 25), magnesium (*n* = 10) and vitamin B12 (*n* = 10). The same supplements were the most frequently mentioned by patients: magnesium (*n* = 23), vitamin D (*n* = 20), and vitamin B12 (*n* = 9) (see Additional File 3 - Figure S2). The most common reasons for taking dietary supplements reported by patients were to improve general health and due to discomfort with muscles, joints or for bone health (Fig. 2).

GPs and patients reported a total of 156 supplements used. Comparing supplement use reported by GPs and their patients demonstrated that 22% (*n* = 35) of the supplements were classified as disclosed (reported by both parties), whereas 78% (*n* = 121) were not disclosed (only reported by one of the parties). GPs were unaware of 82 (53%) supplements taken by 39 (60%) of their patients. 39 (25%) of the supplements were reported by GPs, but not by the patients. Supplements that had a complete match and were reported by both patients and their GPs were vitamin B12, vitamin D and magnesium. Table 2 shows the association between the disclosure of the use of dietary supplements, patient characteristics, and trust in their physician. We did not find evidence for a statistically significant association between supplement disclosure and patient characteristics.

**Table 2:** Association between the disclosure of the use of dietary supplements with patient characteristics and patient trust in their physician (*n* = 123)

	Crude Odds Ratio (95% IC)	*p*-value	Adjusted Odds Ratio (95% CI)	*p*-value ^a^
Female patient (*reference: male*)	2.14 (0.91 to 5.05)	0.081	2.10 (0.81 to 5.48)	0.129
Score trust in the physician ^b^ (*by unit increase*)	0.98 (0.89 to 1.07)	0.599	0.97 (0.88 to 1.08)	0.593
Patients’ higher education (*reference: lower education*) ^c^	0.60 (0.23 to 1.61)	0.311	1.28 (0.44 to 3.72)	0.655
Vitamin/minerals (*reference: other supplements*)	1.86 (0.70 to 4.95)	0.217	1.52 (0.55 to 4.15)	0.418

^a^ Multilevel logistic regression adjusted at GP (ICC = 0.057) and patient (ICC = 0.058) level. Dependent variable: disclosure of dietary supplement, assessed by the match of each supplement reported by patients and their GPs

^b^ Score of the abbreviated Wake Forest Trust in Physician Scale [40]. Score is within 5 to 25, with higher values indicating higher trust

^c^ Secondary School or Third level education *versus* Primary School or None

Of the 45 patients, only five (11%) reported at least one dietary supplement they would be willing to deprescribe if their GP suggested doing so. Of the ten GPs, six (60%) reported at least one dietary supplement they would be willing to deprescribe for 12 of their older patients. In total, patients reported eight dietary supplements that they would be willing to deprescribe, and GPs reported 14 (Additional File 3 - Figure S3). Patients reported they would be willing to deprescribe vitamins and minerals (*n* = 4), herbs (*n* = 2), and fish oil (*n* = 2) (Additional File 3 - Figure S3). GPs reported they would be willing to deprescribe vitamins and minerals (*n* = 6), followed by herbs (*n* = 4), fish oil (*n* = 2), and others (*n* = 2) (Additional File 3 - Figure S3). Looking at patient-GP pairs, there were no matches of dietary supplements chosen for deprescribing reported by both patients and their GPs.

## Discussion

In our sample of GPs from primary care settings in the German-speaking part of Switzerland and a sample of their older patients with polypharmacy, most patients reported taking at least one dietary supplement. The most common reason for taking supplements was to improve general health. GPs seemed to be unaware of most dietary supplements taken by their patients and the disclosure of supplement use was not associated with any patient characteristics. When GPs were asked about their willingness to deprescribe dietary supplements used by their patients, 60% of the GPs reported ≥ 1 supplement they would be willing to stop or reduce. Only 11% of patients reported ≥ 1 supplement they would be willing to stop or reduce. None of the supplements mentioned as potential deprescribing candidates matched in patient-GPs dyads.

In this study, 70% of patients reported to be taking at least one dietary supplement. This rate is higher than in previous studies conducted in Switzerland, which reported the prevalence of supplement use between 26 and 53% [6, 9, 10]. This difference may be due to the fact that these previous studies did not focus on the older population with polypharmacy, and the use of dietary supplements has been associated with older age [6, 7, 32]. In addition, most of the patients in our study reported having good or excellent self-reported health, and it has been reported that people who take dietary supplements have better overall health [14, 39]. The most common reasons for taking dietary supplements were to improve general health and due to discomfort with muscles or joints, or for bone health. Other studies have also reported health maintenance or improvement [7, 13, 38, 39] and bone health [7, 39] as the main reasons for using supplements. However, the comparison with other studies should be considered with caution, as most of the studies on the beliefs and reasons for using dietary supplements did not specifically focus on older adults.

In our sample of older primary care patients with polypharmacy, the most commonly used supplement types were vitamins and minerals, which is in line with previous studies in Switzerland that showed that vitamins and minerals were the most commonly used ones [6, 10, 15]. Specifically, vitamin D, magnesium, and vitamin B12 were the most frequently reported dietary supplements by both patients and GPs. This corroborates the findings of a report published by a Swiss health insurance company, in which vitamin D and vitamin B12 were reported as most frequently used by the Swiss population in 2021 and 2022 [11, 12]. In Switzerland, mandatory health insurance covers select dietary supplements prescribed by physicians, including vitamin D, vitamin B12, and magnesium, and the prescribing of these supplements has been in the last three years [11, 12].

Considering patients’ beliefs towards dietary supplements, most of the patients in our study believed that supplements are beneficial, that they may prevent diseases, and that they are worth the money spent on them. Although in our study patients held generally positive beliefs about dietary supplements, which is in line with previous studies [13, 37, 39], most participants also believed that supplements are not necessary for everyone and that they may interact with other medications. We did not identify any significant difference in beliefs towards dietary supplements among users and non-users. Despite the high use of dietary supplements by the patients in our study, many reported being unsure regarding the risks and benefits of supplements. Other studies have also shown that there is a lack of knowledge regarding dietary supplements, but at the same time there is also an interest in learning more about those [13]. Patients’ decision-making regarding supplement use would benefit from a better understanding of the risks and benefits associated with dietary supplement use.

When comparing dietary supplements reported by GPs and their patients, we found that GPs seemed to be unaware of more than half of the dietary supplements taken by their older patients with polypharmacy. Controversially, most patients believed that they should talk to their GP or another healthcare provider about the use of dietary supplements, and most of the users of dietary supplements responded that they do talk to their GP or pharmacist about taking supplements. Other studies have also reported that patients usually do not disclose their dietary supplement use to their GPs or other health care professionals [5, 14, 44–47]. A systematic review reported that the disclosure rate varied between 12% and 78% [5]. However, most of these studies were conducted in the United States of America, were published before 2010, and collected the disclosure information simply by asking patients whether they disclose the use of dietary supplements to their physicians or healthcare providers or not.

Another interesting finding in our study is that when GPs and patients were asked about the number of prescription medications the patient is currently taking, GPs reported on average two medications more than their patients, and many supplements were reported by GPs, but not by the patients. These findings are worth exploring in future studies, as they likely reflect patients’ medication adherence. The discrepancy in the number of supplements reported by GPs and patients demonstrates that a particular emphasis must be put on establishing adequate medication lists (including supplements) in future medication optimisation efforts.

We did not find any association between supplement disclosure and patient characteristics or trust in the physician. Nevertheless, an association of disclosure of dietary supplements with female gender and education level was reported in other studies [48, 49]. The lack of evidence for an association might be due to the low sample size in our study. In addition, other studies have shown that patient-provider communication plays a role in the disclosure of dietary supplements [41, 46, 47]. Although in our study patients were often unsure about the benefits of dietary supplements, and many understand that supplements may interact with other drugs, many still did not discuss the use of supplements with their GPs, demonstrating a lack of communication between older patients and GPs. Strategies to improve this communication and to optimise medication safety in primary care are thus required.

In our study, the only supplements that were reported by both patients and GPs were vitamin B12, vitamin D and magnesium. These supplements are commonly prescribed in Switzerland, which would explain why GPs are aware of the use of these supplements, but not other over-the-counter supplements. Other studies have identified potential risks of interactions between prescribed medications and dietary supplements when these are used concomitantly [5, 50]. For instance, one patient in our study was taking *St John’s wort*, and two patients were taking *Ginkgo biloba. St John’s wort* is one of the supplements with more risk for interactions with other drugs, and *Ginkgo biloba* poses a high risk of bleeding when used with other medications such as aspirin and warfarin, which are commonly used among older adults [5, 51]. The finding that many supplements are not disclosed reinforces the fact that GPs should actively ask their patients about their use of dietary supplements including non-prescription ones, so that they can identify potential risks, drug-supplement and supplement-disease interactions, and lack of indications [41, 47].

Most of the older patients in our study were not willing to have any dietary supplements deprescribed. This is in line with the results from another study that found that older adults were resistant to having non-prescription medications (including dietary supplements) deprescribed [52]. The low patient willingness to have supplements deprescribed could be explained by the finding of patients’ positive attitudes towards supplements and the overall lack of information regarding dietary supplements. Studies have identified barriers and concerns towards deprescribing prescription medications [53–55]. For instance, patients may be reluctant to change prescription medications that they have been taking for a long time [53–55]. In line with this, patients in our study may also be reluctant to change supplements they have been using for extended periods. Also, patients may feel more “ownership” in taking dietary supplements, as they are easily accessible, which could also pose a barrier to stopping or reducing their use.

Despite the low awareness, most GPs were willing to stop or reduce at least one dietary supplement for at least one of their patients. If they had been aware of all supplements used by patients, they likely would have made even more hypothetical deprescribing suggestions, reinforcing that dietary supplements may be easy targets for discontinuation. Although no previous studies have focused on GP attitudes towards deprescribing specifically dietary supplements, GPs and other health care providers have been found to often suggest dietary supplements as deprescribing targets [28, 33]. When comparing patient and provider attitudes towards deprescribing, none of the supplements chosen by GPs and by patients matched. This discrepancy could be one of the reasons explaining the low implementation rate of deprescribing suggestions in real-world clinical settings. In future research, it will be worthwhile to also compare GP-provider agreement with regards to deprescribing prescription medications.

It is important to note that dietary supplements are important for vulnerable groups at risk for nutritional deficiency, but their use should be individualised and accompanied by a GP, pharmacist, dietitian, or other healthcare professional to avoid interactions and adverse events. Supplements are often used without an indication, can cause harm, and may lead to unnecessary costs. Medications - including supplements - that do not contribute to the patient’s health should be considered for deprescribing to avoid risks and unnecessary costs [25, 56]. However, only when GPs aware of all substances used by their patients, they can provide personalised treatments and make deprescribing recommendations. Our findings shed light on the need to educate GPs to actively ask their patients about the use of dietary supplements when conducting medication optimisation efforts in Swiss primary care settings.

### Strengths and limitations

To the best of our knowledge, this is the first study investigating and comparing older patients’ and GPs’ attitudes towards deprescribing dietary supplements. This study is strengthened by the fact that we collected information directly from patients and their GPs, allowing the comparison of their responses. Although we sought to avoid selection bias by instructing GPs to use a consecutive sampling approach when recruiting patients, we cannot rule out selection bias. At the patient level, we have the limitation that due to feasibility reasons patients could only report a maximum of three supplements in the questionnaire. However, only ten patients (15%) reported three supplements and when piloting the questionnaire, no participant indicated more than three supplements. In addition, in our questionnaire patients only reported whether they were ≥ 65 years old or not, therefore we could not include age in the analysis. We cannot rule out recall bias, as participants may not have reported all their supplements, or volunteer bias, as participants could have been more interested in the study topic compared to non-participants. In addition, we were unable to reach our target sample size, which limits the generalisability of our results. However, our exploratory and hypothesis-generating findings provide valuable insights for future research on the use and deprescribing of dietary supplements in older adults with polypharmacy. The findings of the present study cannot be generalised, given its sample size and restricted location in the German-speaking part of Switzerland. Despite the small sample size, some of our analyses were carried out at the supplement level, allowing a more detailed examination of the dietary supplements disclosed by patients to their GPs. Nevertheless, due to the small sample size, the confidence intervals in the logistic regression model were wide and imprecise.

## Conclusions

GPs in primary care settings in the German-speaking part of Switzerland were not aware of more than half of the dietary supplements used by their older patients with polypharmacy. Older patients seemed to be unsure about the benefits, necessity, and possible risks of dietary supplements and were not willing to have those deprescribed. To optimise the use of medications and dietary supplements by older adults in Swiss primary care, it is crucial for GPs and other healthcare providers to identify which dietary supplements their patients are using. By actively asking patients about their supplement use as part of medication optimisation efforts, healthcare professionals can help reduce medication-related harm.

## Electronic supplementary material

Below is the link to the electronic supplementary material.

Additional File 1: Study questionnaire for patients

Additional File 2: Questionnaire for General Practitioners

Additional File 3: Figure S1: Beliefs about dietary supplements of users and non-users; Table S1: Comparison of agreement on statements on dietary supplements between users and non-users; Figure S2: Dietary supplements used by older patients with polypharmacy living in the German part of Switzerland. Figure S3: Dietary supplements older patients with polypharmacy and their general practitioners would have an interest in deprescribing

## Data Availability

The data that support the findings of this study are not openly available due to reasons of confidentiality. The anonymised data will be made available by the corresponding author for scientific research purposes, after the proposed analysis plan has been approved. Data and documentation will be made available through a secure file exchange platform after approval of the proposal. In addition, a data transfer agreement must be signed (which defines obligations that the data requester must adhere to regarding privacy and data handling). Deidentified participant data limited to the data used for the proposed project will be made available, along with a data dictionary.

## Footnotes

1 In some regions of Switzerland, physicians can dispense medications directly to their patients (self-dispensation cantons).

2 In Switzerland, a pharmacy supplies prescription medication as well as over-the-counter medications and supplements, while drug stores provide non-prescription substances such as personal care products and dietary supplements.
