# Supplementary material for: Exploring GP and patient attitudes towards the use and deprescribing of dietary supplements: a survey study in Switzerland
Source: BMC Prim Care. 2024 Oct 3;25:359. doi: 10.1186/s12875-024-02605-z (PMC11451169; doi:10.1186/s12875-024-02605-z)
Supplement: Supplementary file 1 — Additional File 1: Study questionnaire for patients [file 12875_2024_2605_MOESM1_ESM.pdf]

Exploring GP and patient attitudes towards the use and deprescribing of dietary supplements: a survey study in Switzerland

Renata Vidonscky Lüthold [1,2], Zsofia Rozsnyai [1], Kristie Rebecca Weir [1,3], Sven Streit [1], Katharina Tabea Jungo [1,4,5,*]

[1] Institute of Primary Health Care (BIHAM), University of Bern, 3012 Bern, Switzerland

[2] Graduate School for Health Sciences, University of Bern, 3012 Bern, Switzerland

[3] Sydney School of Public Health, Faculty of Medicine and Health, University of Sydney, 2050 Sydney, Australia

[4] Center for Healthcare Delivery Sciences, Brigham and Women's Hospital, 02115 Boston, MA, United States of America

[5] Division of Pharmacoepidemiology and Pharmacoeconomics, Department of Medicine, Brigham and Women's Hospital and Harvard Medical School, 02115 Boston, MA, United States of America

*Corresponding author: katharina.jungo@protonmail.com

Additional File 1: Study Questionnaire for Patients

Have you already signed the consent form together with your GP?

If you click/check "Yes", you agree to participate.

o Yes (automatic forwarding to the questionnaire)

o No (end of study participation)

1) Questions about the inclusion criteria

1. How old are you (in years)?
   - o 65 years old or older (continue to the next question)
   - o 64 years old or younger (end of study participation)

2. Do you regularly take 5 or more medications? (Regularly means: every day or most days for 30 days or more)
   - o Yes (continue to the next question)
   - o No (end of study participation)

3. Do you live in Switzerland?
    - o Yes (go to next question)
    - o No (end of study participation)

2) Information about your GP

4. What is the name of your general practitioner? Please enter his/her first and last names:

____

5. What is the location of your GPs' office?

____

6. What is the postcode of the practice?

____

7. What is the name of the street where the GP practice is located?

    ____

8. Is this your family doctor? (Definition: if you have a health problem, you usually always consult the same family doctor - except in emergencies

    o Yes (continue with the next question number 9)
    o No (Please go directly to Section 3 Socio-demographic questions)

9. If yes, how long have you been going to this family doctor?

    o 0-9 years
    o 10-19 years
    o 20-29 years
    o 30+ years

3) Socio-demographic questions

We will now ask you some questions in order to better understand who answered our questionnaire.

10. What is your gender?
    - o male
    - o female
    - o other

11. What area do you live in?
    - urban
    - suburban
    - rural

12. What is your postcode?

13. Do you live alone in your household?
    - yes
    - no

14. What is your living situation?
    - Own your house or apartment
    - Rented house or apartment

15. What is your highest completed education?
    - none
    - primary school
    - secondary education (apprenticeship or high school)
    - tertiary education (university or college studies)

16. How do you make ends meet financially?
    - With great difficulty
    - With some difficulty
    - Quite easily
    - Without any problems

17. Where were you born?
    - In the country where I currently live
    - Other country [Please specify country] ______________________

18. What is your first language?
    - Official language of the country where I currently live
    - Other language [Please specify language] ___________________

19. How confident are you in filling out medical forms by yourself?
    - Not at all
    - A little bit
    - Somewhat
    - Quite a bit
    - Extremely

20. Generally speaking, how would you describe your health today?
    - Excellent
    - Very good
    - Good
    - Average
    - Poor

4) Questions about your use of medication

Now we would like to learn more about your experiences with taking medication.

21. I prepare my medication myself:
    - Yes, I prepare and take it myself according to the prescription.
    - No, I receive support in preparing/taking my medication from relatives, the Spitex or at the pharmacy for example.

22. Overall, I am satisfied with my current medications.
    - Strongly agree
    - Agree
    - Don’t know
    - Disagree
    - Strongly disagree

23. How many different kinds of medications do you take regularly? (Regularly means daily or on most days of the week.) Please indicate the number of different kinds of medications.

    Number of different medications: ___________________________________

24. Do you regularly take herbal, vitamin, or mineral supplements?
    - Yes (continue to the next question)

- No (continue to section 5)

25. How many different vitamins, mineral supplements, or herbal medications do you take regularly?

    Number of different dietary supplements: ________________

26. In the last week: What supplements have you taken? Please check all that apply.

    □ Multivitamins
    □ Iron
    □ Calcium
    □ Vitamin A
    □ Vitamin E
    □ Vitamin B12
    □ Vitamin B6
    □ Vitamin C
    □ Vitamin D
    □ Vitamin K
    □ Vitamin B complex
    □ Folic Acid
    □ Magnesium
    □ Zinc
    □ Valerian root
    □ Ginkgo biloba
    □ Turmeric
    □ Echinacea
    □ St. John's wort
    □ Garlic
    □ Ginseng
    □ Omega-3
    □ Chondroitin sulphate
    □ Glucosamine
    □ Other(s): ___________
    ____

5) Questions about your attitude towards and decisions about medication

Now we are going to ask you questions on your thoughts about stopping or reducing the dose of medicines.

27. If my doctor said it was possible I would be willing to stop one or more of my regular medications.
    - Strongly agree
    - Agree
    - Don't know
    - Disagree
    - Strongly disagree

28. I would like to try stopping one of my medications to see how I feel without it.
    - o Strongly agree
    - o Agree
    - o Don't know
    - o Disagree
    - o Strongly disagree

29. Thinking about your current medication list, are there any medications that you would like to stop taking or reduce the dose of?
    - o Yes *(please continue to the next question)*
    - o No, I am not considering stopping or reducing the dose of any medication. *(Please go to Question 31)*

30. In the following table, please state the name(s) of the medication(s) that you would consider stopping or reducing, and the reason why.

Any lines that are not applicable can be left empty.

Name(s) of the medication(s) that you would consider stopping or reducing	Why did you choose this/these medication(s) to stop or reduce?<br><br>*Please check all answers that apply*
Name of the medication:<br><br>___________________________	o It causes side effects.<br>o I do not benefit from it.<br>o I do not like the medication.<br>o The medication is too expensive.<br>o It is inconvenient for me to take this medication.<br>o The tasks involved in taking the medication(s) (e.g. blood glucose monitoring) are stressful for me.<br>o I often forget to take this medication.<br>o Other reason:____________________________
Name of the medication:	o It causes side effects.<br>o I do not benefit from it.<br>o I do not like the medication.

___________________________	○ The medication is too expensive.<br>○ It is inconvenient for me to take this medication.<br>○ The tasks involved in taking the medication(s) (e.g. blood glucose monitoring) are stressful for me.<br>○ I often forget to take this medication.<br>○ Other reason:____________________________
Name of the medication:<br><br>___________________________	○ It causes side effects.<br>○ I do not benefit from it.<br>○ I do not like the medication.<br>○ The medication is too expensive.<br>○ It is inconvenient for me to take this medication.<br>○ The tasks involved in taking the medication(s) (e.g. blood glucose monitoring) are stressful for me.<br>○ I often forget to take this medication.<br>○ Other reason:____________________________
Name of the medication:<br><br>___________________________	○ It causes side effects.<br>○ I do not benefit from it.<br>○ I do not like the medication.<br>○ The medication is too expensive.<br>○ It is inconvenient for me to take this medication.<br>○ The tasks involved in taking the medication(s) (e.g. blood glucose monitoring) are stressful for me.<br>○ I often forget to take this medication.<br>○ Other reason:____________________________

After the table please continue to section 6 “additional questions about stopping medications and your willingness to do so”.

31. You may not want to stop taking a medication or reduce the dose. Here are some reasons why. Which one(s) do you think are the most important reasons for not stopping a medication? (Please select all that apply)

    - ○ The medicine is beneficial.
    - ○ Taking the medicine for a long time so it is better not change it.
    - ○ Taking several medications every day is manageable.
    - ○ The medication does not cause side effects.

- Medication(s) are not expensive.
- Doctors only prescribe medication(s) that are necessary.
- It is easier to take medications than to make healthy lifestyle changes.

Other reasons: ____

32. Do you regularly take vitamins, mineral supplements or herbal medications?
    - Yes
    - No (*If you are not taking such supplements, please go directly. to section 6).*

If the answer is yes, please complete the table below by indicating the three dietary supplements you use most regularly/frequently.

If you are taking other supplements not mentioned above, please consider them as well. We then ask you to answer the following questions for each supplement by ticking the appropriate statements. An example of filling out the table can be found below. (If you are not using supplements, please leave the table blank.)

**Name of the supplement**	**Why do you use this supplement?**	**Who recommended that you take this supplement?**	**I would be willing to stop taking this supplement or reduce its dose.**
Example:<br><br>*Vitamin D____*<br><br>Example	o To improve my general health<br>✖ To strengthen my immune system<br>o For my nerves, mood or stress<br>o For more energy, alertness or mental activity<br>o To improve blood or circulation<br>o To improve sleep<br>o For muscle, joint or bone problems<br>o To regulate body weight or appetite<br>o To improve skin, nails or hair<br>o For complaints due to menopause or prostate<br>o Other reasons: ________________<br>o I don't have a reason.	○ My GP<br>○ Other doctor/specialist<br>○ Pharmacist<br>○ Other health professional<br>○ Relatives<br>○ Friends<br>✖ Myself<br>○ Other (please specify):<br>_________________	○ Strongly disagree<br>○ Disagree<br>✖ Don't know<br>○ Agree<br>○ Strongly agree
**Supplement 1:**<br><br>Name:<br><br>______________	o To improve my general health<br>o To strengthen my immune system<br>o For my nerves, mood or stress<br>o For more energy, alertness or mental activity<br>o To improve blood or circulation<br>o To improve sleep<br>o For muscle, joint or bone problems<br>o To regulate body weight or appetite<br>o To improve skin, nails or hair<br>o For complaints due to menopause or with the prostate<br>o Other reasons: ________________<br>o I don't have a reason.	○ My GP<br>○ Other doctor/specialist<br>○ Pharmacist<br>○ Other health professional<br>○ Relatives<br>○ Friends<br>○ Myself<br>○ Other (please specify):<br>_________________	○ Strongly disagree<br>○ Disagree<br>○ Don't know<br>○ Agree<br>○ Completely agree
**Supplement 2:**<br><br>Name:<br><br>______________	o To improve my general health<br>o To strengthen my immune system<br>o For my nerves, mood or stress<br>o For more energy, alertness or mental activity<br>o To improve blood or circulation<br>o To improve sleep<br>o For muscle, joint or bone problems<br>o To regulate body weight or appetite<br>o To improve skin, nails or hair<br>o For complaints due to menopause or with the prostate<br>o Other reasons: ________________<br>o I don't have a reason.	○ My GP<br>○ Other doctor/specialist<br>○ Pharmacist<br>○ Other health professional<br>○ Relatives<br>○ Friends<br>○ Myself<br>○ Other (please specify):<br>_________________	○ Strongly disagree<br>○ Disagree<br>○ Don't know<br>○ Agree<br>○ Strongly agree

Name of the supplement	Why do you use this supplement?	Who recommended that you take this supplement?	I would be willing to stop taking this supplement or reduce its dose.
**Supplement 3:**<br><br>Name:<br><br>_____________<br>_	o To improve my general health<br>o To strengthen my immune system<br>o For my nerves, mood or stress<br>o For more energy, alertness or mental activity<br>o To improve blood or circulation<br>o To improve sleep<br>o For muscle, joint or bone problems<br>o To regulate body weight or appetite<br>o To improve skin, nails or hair<br>o For complaints due to menopause or with the prostate<br>o Other reasons: _________________<br>o I don't have a reason.	o My GP<br>o Other doctor/specialist<br>o Pharmacist<br>o Other health professional<br>o Relatives<br>o Friends<br>o Myself<br>o Other (please specify): _______________ __	o Strongly disagree<br>o Disagree<br>o Don't know<br>o Agree<br>o Strongly agree

33. Where do you buy the supplements? Select all answer options that apply.

- o Pharmacy
- o Drugstore
- o Supermarket
- o Internet
- o Natural food store
- o In the gym
- o Other location: ____

34. Do you talk to your family doctor or pharmacist about taking supplements?

- o Yes
- o No

<u>6) Additional questions about stopping medication and your willingness to do so:</u>

35. I feel comfortable talking to my doctor about changes to my medication

- o Strongly agree
- o Agree
- o Don't know
- o Disagree
- o Strongly disagree

36. Who would you talk to about stopping or reducing the dose of a medication*? (Please check all that apply)*

- o GP

- Specialist
- Pharmacist
- Family and friends
- Other
    a. Who?

37. What would help you to stop or reduce the dose of a medication? *(Please check all that apply)*
    - A plan or instructions for stopping or reducing the dosage
    - The support of my GP
    - An alternative medication instead
    - An alternative such as a lifestyle change, physiotherapy
    - The option to restart the medicine if I feel I need to, or my symptoms return
    - Other:____

For each of the following, please select the statement that best aligns with your views.

38. What do you think about the medications you take?
    - My medications are important, they keep me alive and help me live well.
    - My medications do what they are supposed to do.
    - I don't really care much about my medications, I take them as my doctor tells me to.

39. How do you get information about your medications?
    - My doctor and I talk about my medications together.
    - I know about my medications – I ask my doctor or read the information leaflet or search online.
    - I don't know much about my medications.

40. How do you make decisions about your medications?
    - I want to be informed, but I trust my doctor to make decisions about my medications.
    - I make decisions about the medications I take, or share the decision with my doctor.
    - Other people (e.g. my doctor or my partner) make decisions for me about my medications.

41. What do you think about the idea of stopping or reducing the dose of one or more of your medications?

- o I would not like to stop any of my medications or reduce the dose.
- o I wish I did not take so many medications and I would stop or reduce the dose of my medications if I could.
- o If my doctor said that it is possible to stop or reduce the dose of a medication that would be ok with me.

7) Questions about your relationship to your family doctor

42. This section is about your relationship with your GP and your trust in them. Please indicate how strongly you agree with each of the statements. There are no right or wrong answers.

	Completely disagree	Disagree	Don’t know	Agree	Completely agree
Sometimes my GP cares more about what is convenient for them than about my medical needs.					
My GP is extremely thorough and careful.					
I completely trust my GP's decision about which medical treatments are best for me.					
My GP is completely honest about the different treatment options available for my health problem.					
All in all, I have complete trust in my GP.					

8) Questions about your use of herbal, vitamin or mineral supplements.

Now we want to hear your opinion about the most common herbal, vitamin or mineral supplements and other dietary supplements (e.g. multivitamins, vitamin D, calcium, valerian, ginkgo biloba, turmeric), even if you don't use any of them.

How much do you agree with the following statements about herbal supplements containing vitamins or minerals?

	Strongly disagree	Disagree	Don't know	Agree	Strongly agree
Supplements can have a positive effect on people's health.					
Supplements can prevent diseases.					
Supplements can cure/treat a disease.					
Supplements are necessary for everyone.					
Supplements can have a negative effect on people's health.					
Supplements are a waste of money.					
Many supplements have not been adequately studied for their efficacy and safety.					
Supplements can interact with prescription drugs.					
I should talk to my GP, pharmacist or other health professional before taking any herbal, vitamin or mineral supplement.					

9) Final questions

Did anyone help you with completing this questionnaire?

- No
- Yes
  - If yes: Who? (please check the answer that applies)
    - Relatives
    - Friends
    - GP
    - GP practice staff
    - Other: ____

You had the opportunity to complete the questionnaire online or on paper. Please confirm that you **only completed one** of the versions of the questionnaire.

- "I confirm that I only completed one of the versions of the questionnaire."

Thank you for your participation, you can now close the survey. If you completed the questionnaire online, you can now close the window. If you completed the questionnaire on paper, please return it to your GP practice as soon as possible.

Thank you for taking the time to complete the questionnaire.

If you have any questions, please do not hesitate to contact us.

Yours sincerely,

Prof. Sven Streit and the rest of the LESS study team
