# Supplementary material for: Exploring GP and patient attitudes towards the use and deprescribing of dietary supplements: a survey study in Switzerland
Source: BMC Prim Care. 2024 Oct 3;25:359. doi: 10.1186/s12875-024-02605-z (PMC11451169; doi:10.1186/s12875-024-02605-z)
Supplement: Supplementary file 2 — Additional File 2: Questionnaire for General Practitioners [file 12875_2024_2605_MOESM2_ESM.pdf]

# Exploring GP and patient attitudes towards the use and deprescribing of dietary supplements: a survey study in Switzerland

Renata Vidonscky Lüthold [1,2], Zsofia Rozsnyai [1], Kristie Rebecca Weir [1,3], Sven Streit [1], Katharina Tabea Jungo [1,4,5,*]

[1] Institute of Primary Health Care (BIHAM), University of Bern, 3012 Bern, Switzerland

[2] Graduate School for Health Sciences, University of Bern, 3012 Bern, Switzerland

[3] Sydney School of Public Health, Faculty of Medicine and Health, University of Sydney, 2050 Sydney, Australia

[4] Center for Healthcare Delivery Sciences, Brigham and Women's Hospital, 02115 Boston, MA, United States of America

[5] Division of Pharmacoepidemiology and Pharmacoeconomics, Department of Medicine, Brigham and Women's Hospital and Harvard Medical School, 02115 Boston, MA, United States of America

*Corresponding author: katharina.jungo@protonmail.com

## Additional File 2: Study Questionnaire for General Practitioners

### Part 1

### GP Profile

***Please complete once as part of this study.***

| Questions about yourself | |
|---|---|
| 1. Name and first name | ______________________________ |
| 2. Address of the practice where you work | ______________________________ |
| 3. Town and postcode of the practice where you work | ______________________________ |
| 4. Location of the practice where you work | *Please check the most appropriate answer.*<br>□ urban<br>□ suburban<br>□ rural |
| 5. Please indicate your gender | □ male<br>□ female<br>□ no answer |
| 6. Please indicate your age (in years): | ______________________________ |

| | |
|---|---|
| 7. What is your first language? | □ German/Swiss German<br>□ French<br>□ Italian<br>□ Other: ______________________ |
| 8. Do you have an FMH title? | □ Yes<br>□ No |
| If yes: Which FMH title do you have? | ______________________ |
| Questions about your daily work | |
| 9. How much experience do you have as a general practitioner? (in years) | ______________________ |
| 10. On how many half-days per week do you see patients? (one half-day equals 10%) | ______________________<br>(please give a number between 1-10) |
| 11. How many consultations do you have on an average workday (this corresponds to two half-days)? | □ <15<br>□ 15-25<br>□ 26-35<br>□ >35 |
| 12. What kind of practice do you work in? | □ Single practice<br>□ Group practice |
| If group practice: How many GPs work in this practice? | ______________________ |
| 13. Before you were invited to participate in this project: Had you ever heard of the concept of deprescribing? | □ Yes<br>□ No |
| General questions about your patients with polypharmacy | |
| 14. Please estimate the percentage of patients in your practice who have polypharmacy (i.e. who regularly take 5 or more medications)? | ______________________<br>(Please enter a number between 0-100) |

| | |
|---|---|
| 15. Please estimate the percentage of patients in your practice who are eligible for stopping or dose reduction? | ________________________________________<br>(Please enter a number between 0-100) |
| 16. For patients taking medications that could potentially be stopped or reduced: What percentage of them have you recommended this to? | ________________________________________<br>(Please enter a number between 0-100) |
| 17. If you did not recommend stopping or reducing the dose of medication, what were the main reasons? | ***Please check all answers that apply.***<br>□ Lack of time<br>□ The medication does not cause any problems.<br>□ The patient wants to continue the medication.<br>□ The patient's symptoms will return when the medication is stopped/reduced.<br>□ Lack of scientific information (or guidelines, etc.) about stopping medication or reducing its dose<br>□ Other reason: ______________________________<br>__________________________________________ |
| Questions about decision-making | |
| 18. How important do you think it is to understand your patients' goals and preferences regarding their medications? | □ not at all important<br>□ a little important<br>□ somewhat important<br>□ pretty important<br>□ really important |
| 19. How often do you talk to your patients about their goals and preferences? | □ never<br>□ rarely<br>□ sometimes<br>□ frequently<br>□ always |
| 20. Please select the option that best reflects how you usually make decisions about stopping a medication or reducing its dose with a patient during your consultation. | □ The patient makes the final decision about stopping or reducing the dose of a medication.<br>□ The patient makes the final decision about stopping a medication or reducing its dose after seriously considering my opinion.<br>□ The patient and I share the responsibility of deciding which medication is best for them. |

| | |
|---|---|
| | □ I make the final decision about stopping a medication or reducing its dose, but seriously consider the patient's opinion.<br>□ I make the final decision about stopping a medication or reducing its dose. |
| 21. Please select the option that best describes how you would like to make decisions about stopping a medication or reducing its dose with a patient in your consultation. | □ The patient makes the final decision about stopping or reducing the dose of a medication.<br>□ The patient makes the final decision about stopping a medication or reducing its dose after seriously considering my opinion.<br>□ The patient and I share the responsibility of deciding which medication is best for them.<br>□ I make the final decision about stopping a medication or reducing its dose, but seriously consider the patient's opinion.<br>□ I make the final decision about stopping a medication or reducing its dose. |

You had the opportunity to choose between filling out an online or a paper questionnaire: Please confirm that you only completed one of the two questionnaires.

o "I confirm that I have only completed one version of the two questionnaires"

Thank you very much for completing this questionnaire. We appreciate you taking the time to do so.

If you have not already done so, we now ask that you complete the other short questionnaires for each of the 5 patients recruited individually.

If you have any questions, please do not hesitate to contact us.

Yours sincerely,

Prof. Sven Streit and the rest of the LESS study team

**Part 2**

**Questions about the hypothetical discontinuation of medications or reduction of their dose in the patients recruited by you**

Please complete one form per patient recruited for this study.

**Procedure:**

1) After you have recruited 5 patients for this study, have their current medication list (digital or on paper) at hand.

2) Then fill out this short questionnaire for all 5 patients and send us their current medication lists (with your comments). You can do this by e-mail or by post

| **Questions about you** | |
|---|---|
| 1. Name and first name | ______________________________________<br>*We need this information in order to be able to assign the participating patients to the participating GPs.* |
| **Questions about the Patient** | |
| 2. Patient's name and first name | ______________________________________ |
| 3. Patient's address | ______________________________________ |
| 4. How long has this patient been your patient? | □ 0-9 years<br>□ 10-9 years<br>□ 20-29 years<br>□ 30+ years |
| **Questions about the patient's use of medication** | |
| 5. How many long-term medications (prescribed for ≥30 days) are currently prescribed for this patient? | ______________________________________<br>Please enter a number. |
| 6. Which long-term medications (prescribed for ≥30 days) are currently prescribed for this patient? | Please take the list of medications you have for this patient.<br>**Mark an X** for all long-term medications (prescribed for ≥30 days).<br>Example: **X** Pantoprazole 20mg, 1x per day |
| 7. Which of these medications do you think are the most important? | Please **circle** them on the medication list.<br>Example: Pantoprazole 20mg, 1x am Tag |

| | | |
|---|---|---|
| 8. Which of these medicines do you think are the least important? | Please **cross them out** on the medication list.<br><br>Example: Pantoprazole 20mg, 1x per day | |
| 9. Would you stop or reduce the dose of any of the medications that the patient is currently taking? | □ Yes<br><br>□ No | |
| 10. If you were to think about stopping or reducing the dose of one of the medicines this patient is currently taking, which would it be? | Please **mark** these medicines with a **circle**.<br><br>Example: Pantoprazole 20mg, 1x per day **O** | |
| 11. Please indicate why you have chosen this/these medication(s) to discontinue or reduce their dose: | Mark all the answers that apply: The medication(s)...<br><br>□ has/have side effects<br><br>□ has/have no benefit<br><br>□ has/have no indication<br><br>□ is/are too expensive<br><br>□ my patient complains about this/these medicine(s)<br><br>□ Other reason:______________________________ | |
| **Questions about taking non-prescription vitamin, mineral, herbal and/or other supplements** | | |
| Such supplements may include iron capsules, fizzy drinks such as Berroca, or drops such as valerian root. Here are some examples: Multivitamins, iron, vitamin D, calcium, valerian root, ginkgo biloba, turmeric. | | |
| 12. Have you ever recommended any supplement to this patient? | □ Yes<br><br>□ No | |
| 13. Do you know if this patient regularly takes supplement? | □ Yes<br><br>□ No<br><br>(including those you did not prescribe) | |
| If yes: Which supplements? | □ Multivitamins<br><br>□ Iron<br><br>□ Calcium<br><br>□ Vitamin A<br><br>□ Vitamin E<br><br>□ Vitamin B12<br><br>□ Vitamin B6<br><br>□ Vitamin C<br><br>□ Vitamin D<br><br>□ Vitamin K<br><br>□ Vitamin B complex<br><br>□ Folic Acid | □ Magnesium<br><br>□ Zinc<br><br>□ Valerian root<br><br>□ Ginkgo biloba<br><br>□ Turmeric<br><br>□ Echinacea<br><br>□ St. John’s wort<br><br>□ Garlic<br><br>□ Ginseng<br><br>□ Omega-3<br><br>□ Chondroitin sulphate<br><br>□ Glucosamine |

| | | | | | |
|---|---|---|---|---|---|
| | | | □ Other(s): ___________ | | |
| 14. Would you stop or reduce any dietary supplement for this patient? | □ Yes<br>□ No | | | | |
| 15. If yes, which dietary supplement? | ____________________________ | | | | |
| Now please go through the statements below and indicate to what extent you agree with them. | | | | | |
| | Strongly disagree | Disagree | Don't know | Agree | Strongly agree |
| This patient tells me everything. | | | | | |
| Sometimes this patient does not follow my recommendations. | | | | | |
| This patient trusts me. | | | | | |
| This patient often disagrees with my recommendations. | | | | | |

You had the opportunity to choose between filling out an online or a paper questionnaire: Please confirm that you only completed one of the two questionnaires.

o "I confirm that I have only completed one version of the two questionnaires"

Thank you for completing this questionnaire. We appreciate you taking the time to do this.

If you have not already done so, we now ask you to fill in the GP profile about yourself and how you work. You only need to complete this once.

If you have any questions, please do not hesitate to contact us.

Yours sincerely,

Prof. Sven Streit and the rest of the LESS study team
