# Supplementary material for: Exploring GP and patient attitudes towards the use and deprescribing of dietary supplements: a survey study in Switzerland
Source: BMC Prim Care. 2024 Oct 3;25:359. doi: 10.1186/s12875-024-02605-z (PMC11451169; doi:10.1186/s12875-024-02605-z)
Supplement: Supplementary file 3 — Additional File 3: Figure S1: Beliefs about dietary supplements of users and non-users; Table S1: Comparison of agreement on statements on dietary supplements between users and non-users; Figure S2: Dietary supplements used by older patients with polypharmacy living in the German part of Switzerland. Figure S3: Dietary supplements older patients with polypharmacy and their general practitioners would have an interest in deprescribing [file 12875_2024_2605_MOESM3_ESM.docx]

**Additional File 3**

**Exploring GP and patient attitudes towards the use and deprescribing of dietary supplements: a survey study in Switzerland**

Renata Vidonscky Lüthold ^1,2^, Zsofia Rozsnyai ^1^, Kristie Rebecca Weir ^1,3^, Sven Streit ^1^, Katharina Tabea Jungo ^1,4,5,*^

^1^ Institute of Primary Health Care (BIHAM), University of Bern, 3012 Bern, Switzerland

^2^ Graduate School for Health Sciences, University of Bern, 3012 Bern, Switzerland

^3^ Sydney School of Public Health, Faculty of Medicine and Health, University of Sydney, 2050 Sydney, Australia

^4^ Center for Healthcare Delivery Sciences, Brigham and Women's Hospital, 02115 Boston, MA, United States of America

^5^ Division of Pharmacoepidemiology and Pharmacoeconomics, Department of Medicine, Brigham and Women's Hospital and Harvard Medical School, 02115 Boston, MA, United States of America

*Corresponding author: [katharina.jungo@protonmail.com](mailto:katharina.jungo@protonmail.com)


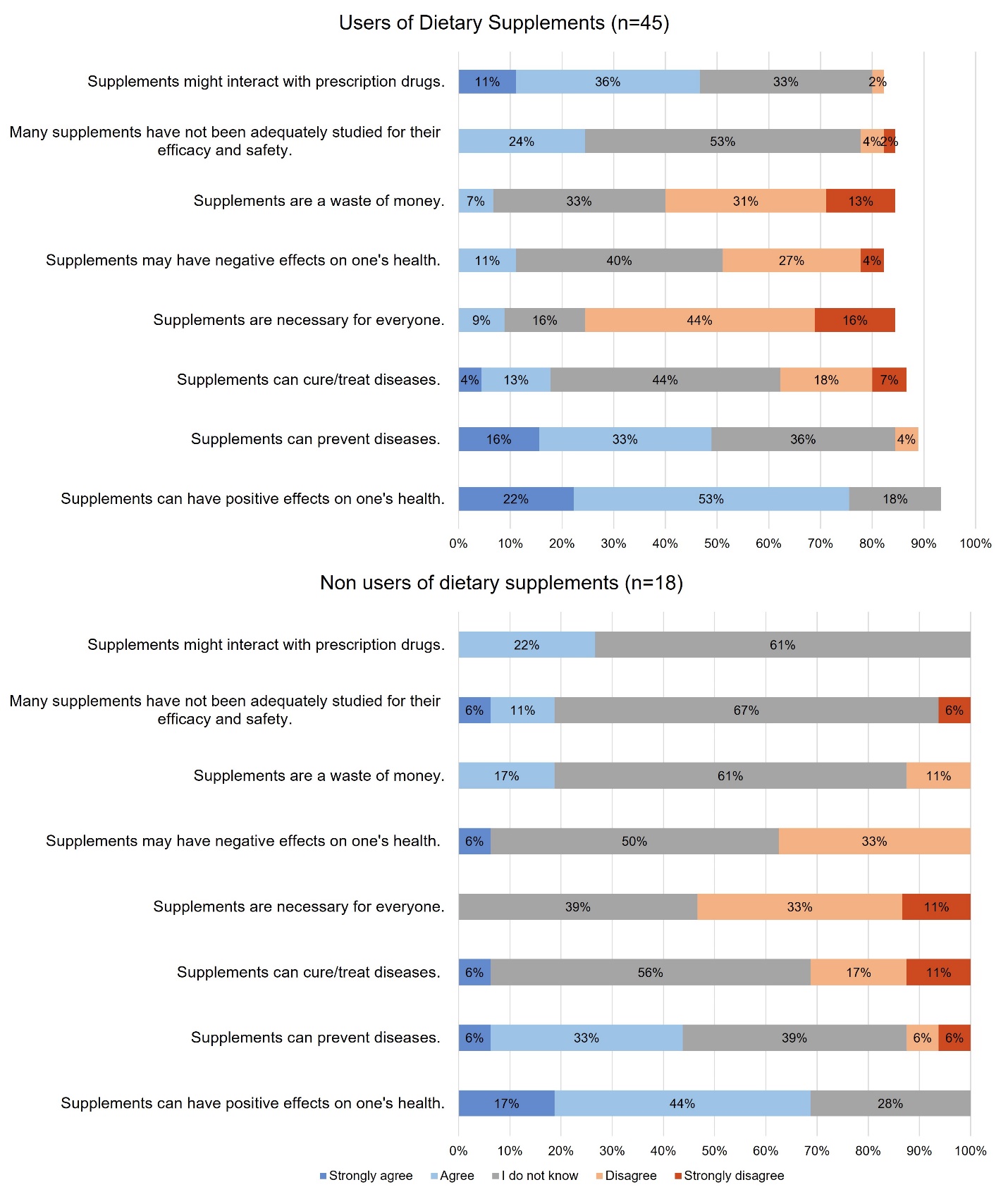


**Figure S1.** Beliefs about dietary supplements of users and non-users (n=65).

**Table S1.** Comparison of agreement on statements on dietary supplements between users and non-users

|  | **Users of dietary supplements (n=45)** | **Non-users of dietary supplements (n=18)** | **p-value** |
| --- | --- | --- | --- |
| Supplements can have positive effects on one's health. | 34 (81%) | 11 (69%) | 0.319 **^a^** |
| Supplements can prevent diseases. | 22 (55%) | 7 (44%) | 0.447 **^a^** |
| Supplements can cure/treat diseases. | 8 (21%) | 1 (6%) | 0.258 **^b^** |
| Supplements are necessary for everyone. | 4 (113%) | 0 (0%) | 0.568 **^b^** |
| Supplements may have negative effects on one's health. | 5 (14%) | 1 (6%) | 0.655 **^b^** |
| Supplements are a waste of money. | 3 (8%) | 3 (19%) | 0.346 **^b^** |
| Many supplements have not been adequately studied for their efficacy and safety. | 11 (30%) | 3 (19%) | 0.515 **^b^** |
| Supplements might interact with prescription drugs. | 21 (57%) | 4 (27%) | 0.068 **^b^** |

Each statement was assessed through 5-point Likert scale questions. “Strongly agree” and “agree” were considered as agreeing with the statement. “I do not know”, “disagree”, and “strongly disagree” were considered as not agreeing.

**^a^** Chi-square test

**^b^** Fischer’s exact test


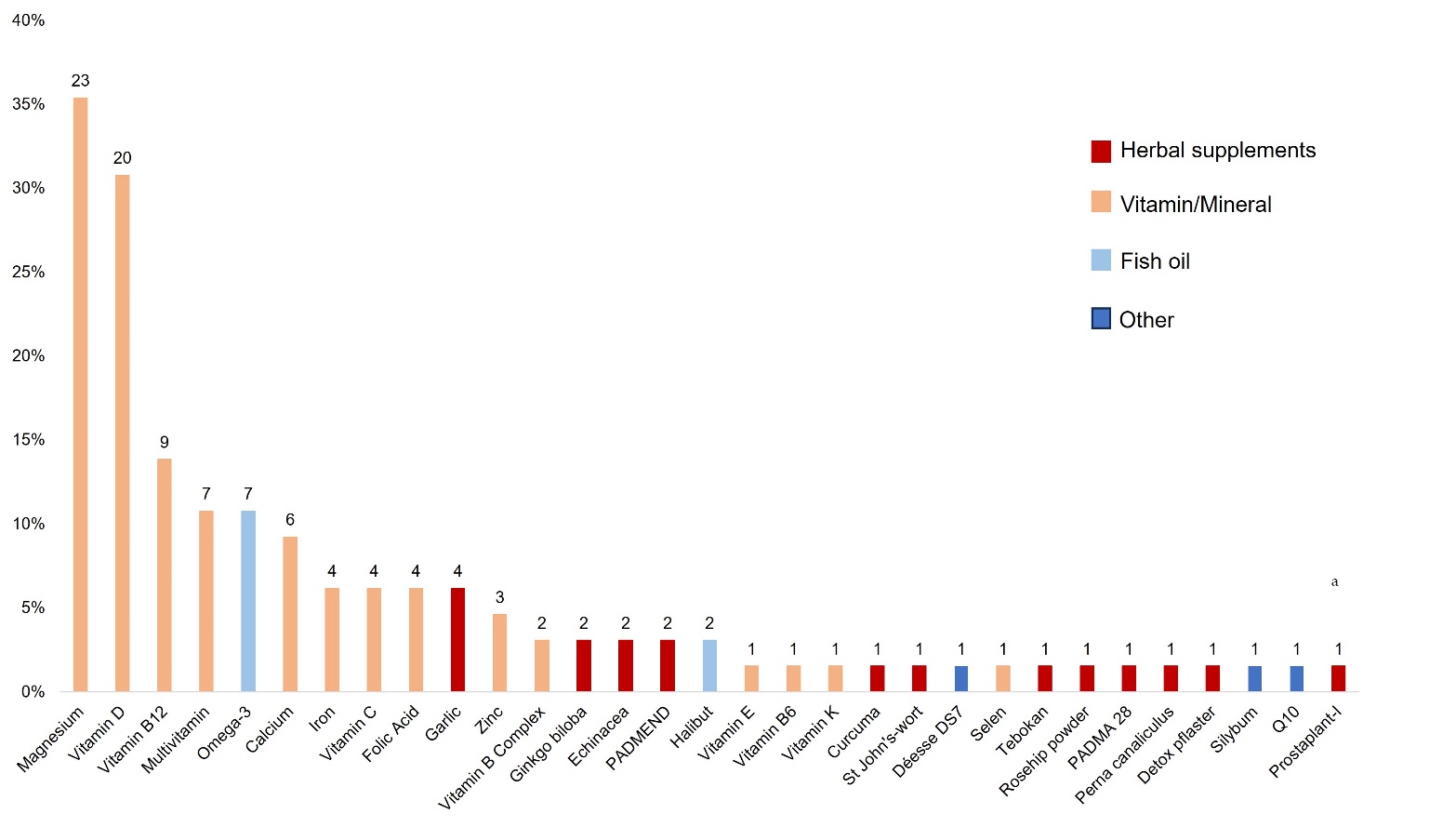


**Figure S2.** Dietary supplements used by older patients with polypharmacy living in the German part of Switzerland (reported by patients).

^a^ Prostaplant-L is a phytotherapeutic product containing herbs.


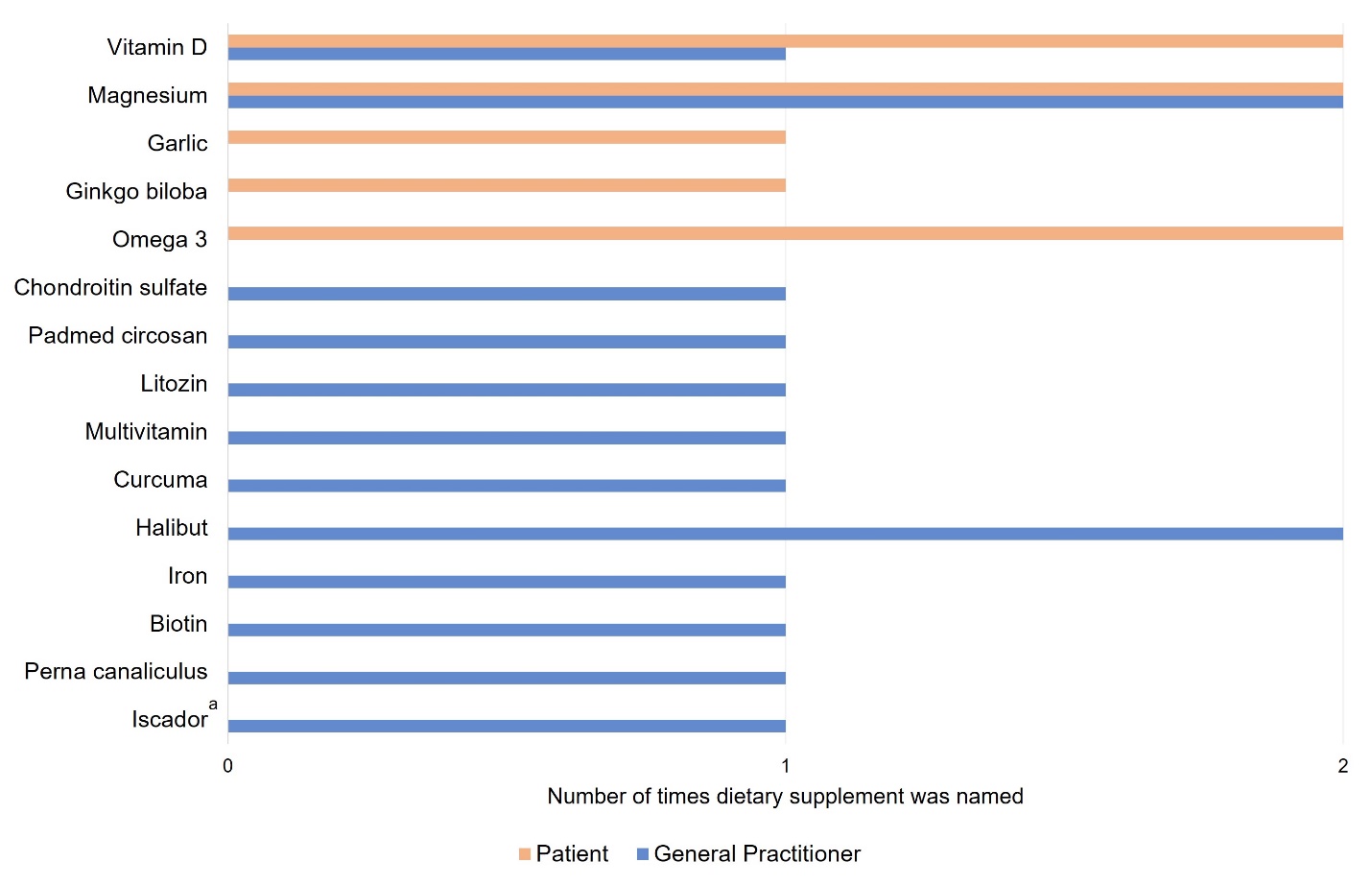


**Figure S3.** Dietary supplements older patients with polypharmacy and their general practitioners would have an interest in deprescribing.

^a^ Iscador is a product containing herbs.
